# Alterations of *slitrk5a* induce OCD-like “checking behaviors” in zebrafish

**DOI:** 10.17912/micropub.biology.001661

**Published:** 2025-07-10

**Authors:** Benjamin A. Sempowski, Kathryn R. Woolford, Jenna Bouhussein, Barbara Lom

**Affiliations:** 1 Biology Department and Neuroscience Program, Davidson College, Davidson, North Carolina, United States; 2 Biology Department, Davidson College, Davidson, North Carolina, United States

## Abstract

The six transmembrane SLITRK proteins differentially regulate important aspects of neuronal development and function. Variants in *SLITRK5* have been associated with complex neuropsychiatric conditions including obsessive compulsive disorder (OCD) and *Slitrk5* knockout mice exhibit overgrooming and anxiety-like behaviors. This study generated *slitrk5a* zebrafish mutants using CRISPR-Cas9. Alterations in *slitrk5a* did not affect F _0_ gross embryonic development or anxiety-like behaviors, however, a repetitive, checking behavior was significantly increased in the novel approach test (NAT). This observation in zebrafish supports an emerging association of *SLITRK5* sequence alterations with OCD-like repetitive behaviors.

**Figure 1. Alterations in slitrk5a increase repetitive, checking behaviors in adult zebrafish without affecting early development or anxiety-associated behaviors:**
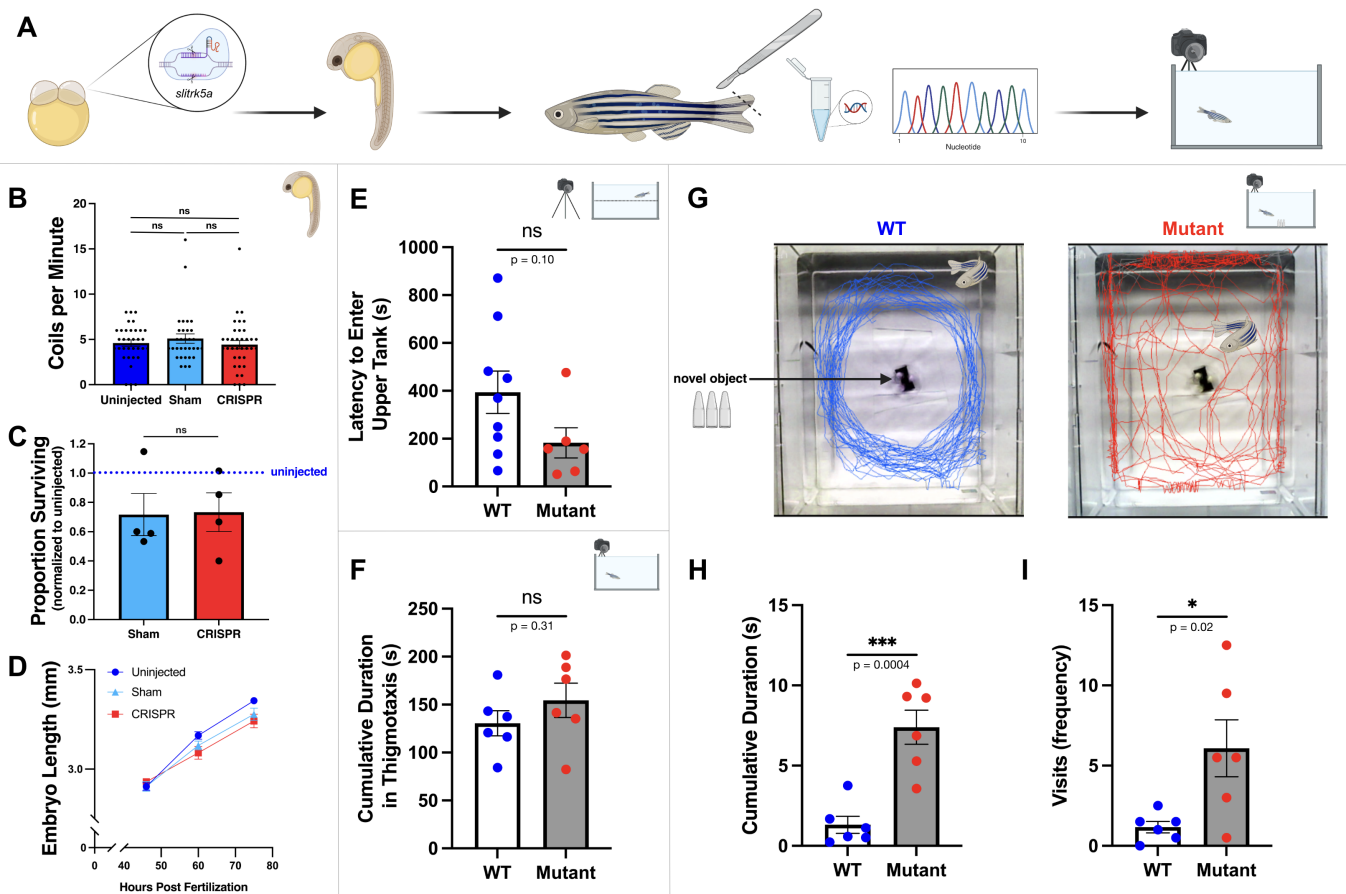
**A)** Zebrafish *(Danio rerio)* embryos were injected with CRISPR-Cas9 reagents to generate *slitrk5a* mutants, then their early development was monitored. After confirmation of induced alterations in *slitrk5a* , repetitive, locomotor, and anxiety-related behaviors were analyzed in adults. **B)** Motor activity, defined as the number of times 24 hpf embryos displayed tail coiling events within the chorion during a one-minute observation period, was similar between uninjected (n=36), sham control (n=34, p=0.71), and CRISPR-injected embryos (n=32, p=0.96) indicating CRISPR reagents did not affect early motor activity levels. **C)** Sham control and CRISPR-injected embryos demonstrated similar mortality rates at 36 hours post fertilization (hpf) compared to uninjected embryos (n=4 trials, p=0.89; normalized to uninjected embryo survival rates) suggesting that CRISPR reagents did not affect survival. **D)** The lengths of uninjected control (n=41), sham control (n=33), and CRISPR-treated embryos (n=30) were not significantly different (p>0.05) at 48, 60, and 75 hpf suggesting CRISPR reagents did not affect early growth rates. **E)** In the novel tank test (NTT), both WT (n=9) and F _0_ *slitrk5a* mutant adults (n=6) demonstrated similar latencies to entering the upper region of the novel tank (p=0.10) suggesting that alterations in *slitrk5a* did not affect this estimate of anxiety. **F)** In the open field (OF) analysis, both WT (n=6) and *slitrk5a* mutant (n=6) adult zebrafish spent similar amounts of time in thigmotaxis, defined as the outer 6 cm edge of the new tank (p=0.31), suggesting that this anxiety-related behavior is also unaffected by alterations in the *slitrk5a* sequence. **G)** Representative swimming tracks during the novel approach test (NAT) for the WT and *slitrk5a* mutant zebrafish. **H-I)** *Slitrk5a* mutants (n=6) spent significantly more time within 5 cm of the central novel object than WT fish (n=6, p=0.0004) and made significantly more visits to the object (p=0.02) suggesting that mutations in *slitrk5a* are associated with increased checking behaviors. Each animal was evaluated in the NTT assay three times and the OF/NAT two times with averages for each individual plotted. All error bars represent standard error of the mean. Graphics created with BioRender.

## Description

The SLITRKs are a family of six transmembrane proteins that play critical roles in central nervous system development and function (Aruga and Mikoshiba, 2003; Won et al., 2019; Puranik and Song, 2024). Structurally, SLITRKs exhibit homology with both the SLIT and TRK protein families that have well-established roles in neuronal development and function. Slit ligands guide axons during early development by signaling through robo transmembrane receptors (Brose and Tessier-Lavigne, 2000; Chedotal, 2007; Blockus and Chedotal, 2016). Transmembrane trk receptors transmit signals from neurotrophin ligands to influence neuronal survival and development (Huang and Reichardt, 2003; Deinhardt and Chao, 2014). SLITRK extracellular domains are characterized by leucine-rich repeats (LRRs) characteristic of molecules that guide synapse formation and stability (de Wit et al., 2011; Ko, 2012; Schroeder and de Wit, 2018) and by intracellular domains that include phosphorylation sites characteristic of trk receptors. SLITRKs are expressed in developing and adult vertebrate neural tissues at synaptic contact sites and have been associated with a variety of human neuropsychiatric conditions such as schizophrenia, obsessive compulsive disorder (OCD), and Tourette syndrome (Proenca et al., 2011; Monteiro and Feng, 2016).

*SLITRK5,* one member of the SLITRK family, plays diverse roles in regulating central nervous system processes including neurite outgrowth, synapse formation, dendritic branching, and signal transmission (Yim et al., 2013; Um et al., 2014; Song et al., 2015; Kang et al., 2016; Puranik and Song, 2024). *Slitrk5* is expressed in regions of the developing central nervous system during periods of morphogenesis and synaptogenesis and is highly expressed in the adult CA1 region of the hippocampus, occipital and frontal lobes of the brain, spinal cord, and medulla, indicating a plausible association with multiple areas of neuron growth and central nervous system processing (Aruga et al., 2003; Beaubien and Cloutier, 2009; Meyer, 2014; Round et al., 2014). Variants and epigenetic modifications of human *SLITRK5* have been linked to behavioral and neurodevelopment conditions including obsessive-compulsive disorder (OCD), Tourette syndrome, attention deficit/hyperactivity disorder (ADHD), conduct disorder (CD), and schizophrenia (Proenca et al., 2011; Song et al., 2017; Salesse et al., 2020; Halvorsen et al., 2021; Chiocchetti et al., 2022; Puranik and Song, 2024). Additionally, *Slitrk5* knockout (KO) mice exhibit severe anxiety and OCD-like behaviors such as excessive and harmful self-grooming (Shmelkov et al., 2010). Physiologically, elevated activity in the orbitofrontal cortex has also been observed, consistent with functional imaging findings in humans with OCD, implicating dysregulation of corticostriatal circuitry (Rauch et al., 2007; Ting and Feng, 2011). Increased SLITRK5 is also associated with epilepsy in a rat model (Liu et al., 2023).

The zebrafish ( *Danio rerio* ) is a cost-effective, genetically accessible, and easily manipulable model system to study nervous system development and neuropsychiatric conditions (Fetcho and Liu, 1998; Stewart et al., 2015; Fontana et al., 2018; Nelson and Granato, 2022). *Slitrk5a* expression in the developing zebrafish, first detected at 48 hours post fertilization (hpf), is observed in the retina, midbrain, medulla oblongata, valvula cerebelli, pituitary gland, and spinal cord suggesting *slitrk5a* could contribute to neurogenesis, morphogenesis, and/or synaptogenesis (Round et al., 2014). This study specifically investigated *slitrk5a* in the zebrafish central nervous system with downstream implications on behaviors associated with neuropsychiatric conditions such as obsessive compulsive disorder (OCD).

To generate mutant zebrafish with altered *slitrk5a* gene sequences, CRISPR-Cas9 reagents were microinjected into embryonic zebrafish at the single-cell stage ( Fig. 1A ; Hwang et al., 2013; Sorlien et al., 2018). The embryos were examined in early development then grown to adulthood when they could be genotyped to determine if the *slitrk5a* sequence had been altered. CRISPR-injected, sham-injected control, and uninjected control embryos at 24 hpf displayed similar (p>0.05) average tail coiling events per minute indicating that CRISPR reagents did not alter early motor activity ( Fig. 1B ). At 36 hpf, CRISPR-injected and sham-injected control survived at similar rates (p=0.89) suggesting that CRISPR reagents did not alter survival rates ( Fig. 1C ). The reduced survival rates of injected (sham- and CRISPR-injected) embryos compared to uninjected control embryos are not unexpected given that microinjection necessarily damages the chorion. Embryo lengths ( Fig. 1D ) were also similar (p>0.05) in all comparisons of uninjected (n=41), sham-injected (n=33), and CRISPR-injected (n=30) embryos at 48, 60, and 75 hpf, indicating that gross morphological growth rates were unaffected by microinjection, CRISPR reagents, or the resulting genetic alterations. CRISPR-Cas9 editing of a target gene was not expected to occur successfully in all cells of all embryos. Consequently, changes in *slitrk5a* sequence could cause growth alterations that are not discernible in an F _0_ population prior to genotyping.

Out of a cohort of approximately 50 CRISPR-injected embryos, 30 viable adult fish resulted, six of which were identified as having alterations in the *slitrk5a* gene sequence on chromosome 15. Three behavioral assays were used to compare adult zebrafish behavior in wild type (WT) and *slitrk5a* mutant F _0_ fish reared from the same clutch of embryos. The novel tank test (NTT) was administered ( Fig. 1E ) to estimate levels of anxiety in zebrafish through their latency to enter the upper half of the new tank (Kysil et al., 2017; Duarte et al., 2019). Typically, fish immediately swim to the bottom of a new tank, begin to explore as they acclimate, and eventually enter the upper portions of the tank, swimming toward the surface. The latency for the fish to swim toward the surface of the water can be used as an estimate for anxiety (Kysil et al., 2017). No significant difference (p=0.10) was observed between latencies for WT (n=9) and mutant fish (n=6) to enter the upper half of the novel tank, suggesting that altering the *slitrk5a* sequence did not affect this measure of anxiety.

An open field (OF) assay (Johnson and Hamilton, 2017) was used to assess general swimming behavior and thigmotaxis, the tendency of the fish to swim close to the walls of the tank, an anxiety-related behavior. No significant difference was observed in thigmotaxis ( Fig. 1F ) between the WT controls (n=6) and *slitrk5a* mutant adult fish (n=6) during open field observation (p=0.23), suggesting alterations in the *slitrk5a* sequence also did not alter this anxiety-related behavior. Moreover, the OF assay revealed no significant differences between the mutant fish and WT controls in average swimming velocity (mutant: 8.8 + 0.96 cm/s; WT: 7.9 + 0.70 cm/s; p=0.45) or total distance swum (mutant: 2510 + 268 cm; WT: 2303 + 203 cm; p=0.55) indicating that mutations in the *slitrk5a* sequence did not alter general swimming ability or behavior.

Finally, the novel approach test (NAT) was used to assess anxiety-like and OCD-like behaviors. In the NAT, fish are placed into a tank with a novel object to model fear as the fish may perceive the new object as potentially harmful (Johnson and Hamilton, 2017; Hamilton et al., 2017). The path of WT fish demonstrated a consistent peripheral circling patterns with few interactions with the central novel object whereas the paths of *slitrk5a* mutant fish demonstrated repeated approaches to the novel object, illustrating an OCD-like repetitive checking behavior ( Fig. 1G ). A significant increase in the overall number of visits to the novel object was observed ( Fig. 1H ; p=0.0004) as well as a significant increase in the amount of time spent around the novel object in *slitrk5a* mutant fish compared to WT fish ( Fig. 1I ; p=0.02). These data suggest that alterations in *slitrk5a* induced repetitive, OCD-like behaviors in zebrafish. In the NAT, *slitrk5a* mutant zebrafish interacted significantly more with the novel object; this checking behavior is consistent with the loss of *SLITRK5* in mice that led to an OCD-like repetitive behavior of overgrooming (Schmelkov et al., 2010). Additionally, this observation parallels human behavior as some individuals with OCD repetitively check stimuli or objects that could potentially pose a threat even after confirming that no threat exists (APA, 2013).

The creation of F _0_ *slitrk5a* mutant zebrafish demonstrates feasibility for CRISPR-Cas9 gene targeting to create mutant fish in which developmental and behavioral analyses can be conducted to examine the roles of *slitrk5a* in neuronal development and behavior. That a behavioral phenotype of significantly enhanced checking was observed in a small number of likely mosaically edited F _0_ fish in this preliminary study warrants investment in future studies to create a homozygous loss-of-function *slitrk5a* mutant line of zebrafish for analysis of development and behavior.

## Methods

**Zebrafish:** This study followed protocols approved by the Davidson College Animal Care and Use Committee. Adults were housed in a recirculating aquatic system kept at a 28 °C with a 14:10 hour light:dark cycle. Adult fish were fed twice daily with brine shrimp hatched in-house and Aquafeed Z Gel Cubes. Embryos and early larvae were raised in 10 cm Petri dishes with system water supplemented with 0.0005% methylene blue as a gentle antibiotic (Nüsslein-Volhard and Dahm, 2002). Debris and dead embryos were removed daily and the water was replaced. Larvae and juvenile fish were reared in 750 mL plastic beakers with fish system water plus 0.0005% methylene blue until they were able to feed on brine shrimp (typically ~14 days). Water changes were completed every other day and fish were closely monitored until they had grown sufficiently to be moved into recirculating colony system tanks.

**Generation of *slitrk5a* Mutants Using CRISPR-Cas9:** A CRISPR crRNA was designed to target the beginning of exon one of the zebrafish *slitrk5a* gene using Integrated DNA Technologies’ (IDT) design portal to maximize specificity and minimize off-target effects. Upon receipt, the Alt-R S.p. Cas9 Nuclease V3 was diluted to 57 μM in Cas9 buffer (20 mM Tris-HCl, 600 mM KCl, 20% glycerol) as previously described (Sorlien et al., 2018; Wu et al., 2018). To form the gRNAs, equal amounts of crRNA and trans-activating (tracr) RNA were mixed, diluted to 57 μM in duplex buffer and annealed by heating to 95 °C for five minutes then cooled on ice. To generate the ribonucleoprotein complex (RNP), equal volumes of annealed gRNAs and Cas9 solutions were mixed, incubated at 37 °C for five minutes then cooled on ice, generating a 28.5 μM RNP solution. Approximately 1 nL of RNP was microinjected into zebrafish embryos at the single-cell stage. The resulting embryos were closely monitored throughout development and reared to adults.

**Early Developmental Analysis:** Wild-type (AB background) zebrafish were injected with CRISPR-Cas9 reagents targeting *slitrk5a* as described above (CRISPR treatment). In parallel, a cohort of embryos from the same clutch were similarly microinjected with ~1 nL of 0.1% phenol red (sham control) and a cohort of embryos were left untreated (uninjected control). To assess for potential developmental differences, larvae were imaged with a Nikon SMZ1270 stereomicroscope equipped with a digital camera. Early embryonic motor activity was measured by counting the number of spontaneous tail coiling events per minute while embryos were in the chorion at 24 hpf with the observer unaware of the treatment group (de Oliveira et al., 2021; von Hellfield et al., 2023). Tail coiling events were manually scored as visible contractions of the animal's tail during a one minute observation. As a general indication of development, body lengths (anterior-posterior) of embryos at 48, 60, and 75 hpf were measured without knowledge of the treatment group using ImageJ. Additionally, 36 hpf survival rates across the three groups of embryos were measured for four separate experiments.

**gDNA Isolation and Genotyping of Adult Zebrafish:** At approximately three months of age, samples were obtained of each adult’s caudal fin as previously described (Westerfield, 2007). Genomic DNA (gDNA) was extracted from the tissue using the Qiagen DNeasy Blood & Tissue Kit following manufacturer specifications. Isolated gDNA was then used to amplify the genomic region around *slitrk5a* via standard polymerase chain reaction (PCR) *.* PCR products were purified using the Zymo DNA Clean & Concentrate Kit and then sequenced with Sanger sequencing via Eurofins Genomics. Sequences were aligned to the zebrafish *slitrk5a* reference sequence using SnapGene. Sequencing alignments were analyzed for mutations indicative of non-homologous end-joining (NHEJ) DNA repair initiated by CRISPR-guided Cas9 double-stranded (DS) breaks in the region targeted by the gRNA. A non-targeted portion of the *slitrk5a* gene adjacent to the target site remained intact with nearly 100% homology to the reference sequence. Mutant individuals with alterations in the *slitrk5a* sequence were then housed separately from WT adults with intact *slitrk5a* sequences within the colony where their caudal fins regenerated before behavioral testing.

**Novel Tank Test (NTT):** The novel tank test (NTT) was administered as previously described to record the latency for isolated adult fish to enter the upper half of a new tank (Kysil et al., 2017; Duarte et al., 2019). Zebrafish were removed from colony tanks, individually placed into 2.0 L transport tanks, and allowed to acclimate for one hour. Acclimatized fish were then individually placed into a 9.5 L tank (32.5 x 21 x 17.5 cm) and video recorded for 10 minutes using EthoVision XT tracking software. If a fish did not enter the upper half of the tank after ten minutes, they were removed and returned to their individual 2.0 L transport tank. The initial latency for the fish to enter to the upper half of the tank was analyzed using EthoVision XT software. Each individual fish was observed in the NTT assay three times.

**Open Field (OF) Observation and the Novel Approach Test (NAT):** Open field (OF) observations and the novel approach test (NAT) were performed as previously described (Hamilton et al., 2017; Johnson and Hamilton, 2017). Fish were removed from colony tanks and individually placed into 2.0 L transport tanks, with enrichment and allowed to acclimate for one hour. Fish were then placed individually into a 21 L tank (37.5 x 31 x 17.5 cm) and video recorded for five minutes. This five minute acclimation period served as the open field (OF) observation. Subsequently, a novel object (three yellow, plastic 1.5 mL tubes glued to a 60 mm plastic petri dish lid) was placed in the center of the tank and observation continued for an additional five minutes. Internal EthoVision XT analysis was performed to calculate the dependent variables in this study. Thigmotaxis was defined as the peripheral region of the tank within 6 cm of the edges on all four sides. Visits to the novel object were defined as the fish approaching the central object within 5 cm. Each individual fish was observed on the OF/NAT assay twice.

**Statistical Analysis:** Statistical analysis was performed using GraphPad Prism 10. Survival comparisons used paired t-tests. Motor activity and embryo length used ANOVA multivariate analysis. Comparisons for all three behavioral analyses (NAT, NTT, OF) used unpaired t-tests. A p-value of ≤0.05 was considered significant (*) in all analyses.

## Reagents

**Table:** 

**Reagent/Instrument**	**Source**	**Catalog/Identifier**
Agarose	Fisher Scientific	BP165
Alt-R™ CRISPR-Cas9 crRNA	Integrated DNA Technologies	5’ - GGTTCCTCAAGGAGACCTCC - 3’
Alt-R™ S.p. Cas9 Nuclease V3	Integrated DNA Technologies	1081058
Alt-R™ CRISPR-Cas9 tracrRNA	Integrated DNA Technologies	1072532
Aquafeed Z-cubes	Clear H2O	60-01-0250
Brine shrimp eggs	Bio-Marine	Artemia cysts
DNA Clean & Concentrator-100 Kit	Zymo	D4029
DNeasy Blood & Tissue Kit	Qiagen	69504
Duplex buffer	Integrated DNA Technologies	11-01-03-01
Methylene blue	Thermo Scientific	414240250
OneTaq 2X Master Mix	New England Biolabs	M0482S
Sequencing	Eurofins Genomics	n/a
Software - behavior analysis	Noldus	EthoVision XT 16
Software - graphing and statistics	GraphPad	Prism 10.4.1
Software - image analysis	Public domain	ImageJ
Software - sequence analysis	Dotmatics	SnapGene
Stereomicroscope	Nikon	SMZ1270
Tricaine methanesulfonate	Western Chemical	Tricaine-S
Primer - PCR forward	5’ - GGTAGTCCGGCTCTATTTGAAG - 3’	
Primer - PCR reverse	5’ - GGCTTGTTTGTGGTGGTAATG - 3’	
Primer - sequencing	5’ - TTGGCATCGTACCATAAAGCATAG - 3’	

## References

[R1] American Psychological Association. 2013. Section II: Obsessive-compulsive and related disorders. Diagnostic and Statistical Manual of Mental Disorders, 5th Edition. Arlington, VA: American Psychiatric Association.

[R2] Aruga J, Mikoshiba K (2003). Identification and characterization of Slitrk, a novel neuronal transmembrane protein family controlling neurite outgrowth.. Mol Cell Neurosci.

[R3] Aruga J, Yokota N, Mikoshiba K (2003). Human SLITRK family genes: genomic organization and expression profiling in normal brain and brain tumor tissue.. Gene.

[R4] Beaubien F, Cloutier JF (2009). Differential expression of Slitrk family members in the mouse nervous system.. Dev Dyn.

[R5] Blockus H, Chédotal A (2016). Slit-Robo signaling.. Development.

[R6] Brose K, Tessier-Lavigne M (2000). Slit proteins: key regulators of axon guidance, axonal branching, and cell migration.. Curr Opin Neurobiol.

[R7] Chédotal A (2007). Slits and their receptors.. Adv Exp Med Biol.

[R8] Chiocchetti AG, Yousaf A, Waltes R, Bernhard A, Martinelli A, Ackermann K, Haslinger D, Rotter B, Krezdorn N, Konrad K, Kohls G, Vetro A, Hervas A, Fernández-Rivas A, Freitag CM (2022). The methylome in females with adolescent Conduct Disorder: Neural pathomechanisms and environmental risk factors.. PLoS One.

[R9] de Wit J, Hong W, Luo L, Ghosh A (2011). Role of leucine-rich repeat proteins in the development and function of neural circuits.. Annu Rev Cell Dev Biol.

[R10] Deinhardt K, Chao MV (2014). Trk receptors.. Handb Exp Pharmacol.

[R11] de Oliveira AAS, Brigante TAV, Oliveira DP. 2021. Tail coiling assay in zebrafish ( *Danio rerio* ) embryos: Stage of development, promising positive control candidates, and selection of an appropriate organic solvent for screening of developmental neurotoxicity (DNT). Water. 13: 119

[R12] Duarte T, Fontana BD, Müller TE, Bertoncello KT, Canzian J, Rosemberg DB (2019). Nicotine prevents anxiety-like behavioral responses in zebrafish.. Prog Neuropsychopharmacol Biol Psychiatry.

[R13] Fetcho JR, Liu KS (1998). Zebrafish as a model system for studying neuronal circuits and behavior.. Ann N Y Acad Sci.

[R14] Fontana BD, Mezzomo NJ, Kalueff AV, Rosemberg DB (2017). The developing utility of zebrafish models of neurological and neuropsychiatric disorders: A critical review.. Exp Neurol.

[R15] Halvorsen M, Samuels J, Wang Y, Greenberg BD, Fyer AJ, McCracken JT, Geller DA, Knowles JA, Zoghbi AW, Pottinger TD, Grados MA, Riddle MA, Bienvenu OJ, Nestadt PS, Krasnow J, Goes FS, Maher B, Nestadt G, Goldstein DB (2021). Exome sequencing in obsessive-compulsive disorder reveals a burden of rare damaging coding variants.. Nat Neurosci.

[R16] Hamilton TJ, Morrill A, Lucas K, Gallup J, Harris M, Healey M, Pitman T, Schalomon M, Digweed S, Tresguerres M (2017). Establishing zebrafish as a model to study the anxiolytic effects of scopolamine.. Sci Rep.

[R17] Huang EJ, Reichardt LF (2003). Trk receptors: roles in neuronal signal transduction.. Annu Rev Biochem.

[R18] Hwang WY, Fu Y, Reyon D, Maeder ML, Tsai SQ, Sander JD, Peterson RT, Yeh JR, Joung JK (2013). Efficient genome editing in zebrafish using a CRISPR-Cas system.. Nat Biotechnol.

[R19] Johnson A, Hamilton TJ (2017). Modafinil decreases anxiety-like behaviour in zebrafish.. PeerJ.

[R20] Kang H, Han KA, Won SY, Kim HM, Lee YH, Ko J, Um JW (2016). Slitrk Missense Mutations Associated with Neuropsychiatric Disorders Distinctively Impair Slitrk Trafficking and Synapse Formation.. Front Mol Neurosci.

[R21] Ko J (2012). The leucine-rich repeat superfamily of synaptic adhesion molecules: LRRTMs and Slitrks.. Mol Cells.

[R22] Kysil EV, Meshalkina DA, Frick EE, Echevarria DJ, Rosemberg DB, Maximino C, Lima MG, Abreu MS, Giacomini AC, Barcellos LJG, Song C, Kalueff AV (2017). Comparative Analyses of Zebrafish Anxiety-Like Behavior Using Conflict-Based Novelty Tests.. Zebrafish.

[R23] Liu Y, Zhang L, Ai M, Xia D, Chen H, Pang R, Mei R, Zhong L, Chen L (2023). Upregulation of SLITRK5 in patients with epilepsy and in a rat model.. Synapse.

[R24] Meyer MA (2014). Highly Expressed Genes within Hippocampal Sector CA1: Implications for the Physiology of Memory.. Neurol Int.

[R25] Monteiro P, Feng G (2015). Learning From Animal Models of Obsessive-Compulsive Disorder.. Biol Psychiatry.

[R26] Nelson JC, Granato M (2022). Zebrafish behavior as a gateway to nervous system assembly and plasticity.. Development.

[R27] Nüsslein-Volhard C, Dahm R. 2002. Zebrafish: A Practical Approach. New York: Oxford University Press.

[R28] Proenca CC, Gao KP, Shmelkov SV, Rafii S, Lee FS (2011). Slitrks as emerging candidate genes involved in neuropsychiatric disorders.. Trends Neurosci.

[R29] Puranik N, Song M (2024). Insight into the Association between Slitrk Protein and Neurodevelopmental and Neuropsychiatric Conditions.. Biomolecules.

[R30] Rauch SL, Wedig MM, Wright CI, Martis B, McMullin KG, Shin LM, Cannistraro PA, Wilhelm S (2006). Functional magnetic resonance imaging study of regional brain activation during implicit sequence learning in obsessive-compulsive disorder.. Biol Psychiatry.

[R31] Round J, Ross B, Angel M, Shields K, Lom B (2013). Slitrk gene duplication and expression in the developing zebrafish nervous system.. Dev Dyn.

[R32] Salesse C, Charest J, Doucet-Beaupré H, Castonguay AM, Labrecque S, De Koninck P, Lévesque M (2020). Opposite Control of Excitatory and Inhibitory Synapse Formation by Slitrk2 and Slitrk5 on Dopamine Neurons Modulates Hyperactivity Behavior.. Cell Rep.

[R33] Schroeder A, de Wit J (2018). Leucine-rich repeat-containing synaptic adhesion molecules as organizers of synaptic specificity and diversity.. Exp Mol Med.

[R34] Shmelkov SV, Hormigo A, Jing D, Proenca CC, Bath KG, Milde T, Shmelkov E, Kushner JS, Baljevic M, Dincheva I, Murphy AJ, Valenzuela DM, Gale NW, Yancopoulos GD, Ninan I, Lee FS, Rafii S (2010). Slitrk5 deficiency impairs corticostriatal circuitry and leads to obsessive-compulsive-like behaviors in mice.. Nat Med.

[R35] Song M, Giza J, Proenca CC, Jing D, Elliott M, Dincheva I, Shmelkov SV, Kim J, Schreiner R, Huang SH, Castrén E, Prekeris R, Hempstead BL, Chao MV, Dictenberg JB, Rafii S, Chen ZY, Rodriguez-Boulan E, Lee FS (2015). Slitrk5 Mediates BDNF-Dependent TrkB Receptor Trafficking and Signaling.. Dev Cell.

[R36] Song M, Mathews CA, Stewart SE, Shmelkov SV, Mezey JG, Rodriguez-Flores JL, Rasmussen SA, Britton JC, Oh YS, Walkup JT, Lee FS, Glatt CE (2017). Rare Synaptogenesis-Impairing Mutations in SLITRK5 Are Associated with Obsessive Compulsive Disorder.. PLoS One.

[R37] Sorlien EL, Witucki MA, Ogas J (2018). Efficient Production and Identification of CRISPR/Cas9-generated Gene Knockouts in the Model System Danio rerio.. J Vis Exp.

[R38] Stewart AM, Ullmann JF, Norton WH, Parker MO, Brennan CH, Gerlai R, Kalueff AV (2014). Molecular psychiatry of zebrafish.. Mol Psychiatry.

[R39] Ting JT, Feng G (2011). Neurobiology of obsessive-compulsive disorder: insights into neural circuitry dysfunction through mouse genetics.. Curr Opin Neurobiol.

[R40] Um JW, Kim KH, Park BS, Choi Y, Kim D, Kim CY, Kim SJ, Kim M, Ko JS, Lee SG, Choii G, Nam J, Heo WD, Kim E, Lee JO, Ko J, Kim HM (2014). Structural basis for LAR-RPTP/Slitrk complex-mediated synaptic adhesion.. Nat Commun.

[R41] von Hellfeld R, Gade C, Baumann L, Leist M, Braunbeck T (2023). The sensitivity of the zebrafish embryo coiling assay for the detection of neurotoxicity by compounds with diverse modes of action.. Environ Sci Pollut Res Int.

[R42] Westerfield M. 2007. The Zebrafish Book. A Guide for the Laboratory Use of Zebrafish ( *Danio rerio* ), 5th Edition. Eugene, OR: University of Oregon Press.

[R43] Won SY, Lee P, Kim HM (2019). Synaptic organizer: Slitrks and type IIa receptor protein tyrosine phosphatases.. Curr Opin Struct Biol.

[R44] Wu RS, Lam II, Clay H, Duong DN, Deo RC, Coughlin SR (2018). A Rapid Method for Directed Gene Knockout for Screening in G0 Zebrafish.. Dev Cell.

[R45] Yim YS, Kwon Y, Nam J, Yoon HI, Lee K, Kim DG, Kim E, Kim CH, Ko J (2013). Slitrks control excitatory and inhibitory synapse formation with LAR receptor protein tyrosine phosphatases.. Proc Natl Acad Sci U S A.

